# Learning to Pay Attention

**DOI:** 10.1371/journal.pbio.0050166

**Published:** 2007-05-08

**Authors:** Rachel Jones

Our sensory system is constantly bombarded with inputs, but owing to the brain’s finite processing power, we are forced to pay attention to only a tiny proportion of these inputs at any given time. In a new study, Richard Davidson and colleagues report that intensive training in meditation can alter the way in which the brain allocates attentional resources to important stimuli, allowing people to improve their performance on a demanding visual task.

In the “attentional blink” task, volunteers were asked to identify two “target” stimuli—for example, two particular numbers—in a stream of rapidly presented “non-target” stimuli—for example, letters—which are irrelevant to the task. When the first target number appears on the screen, it captures the attention of the subject, and this can prevent the person from spotting the second target if it appears within around half a second of the first (the attentional blink). It is as if the brain is so busy processing the first target that it can’t also process the second, and therefore the second target goes unnoticed. However, the attentional blink does not represent a structural processing bottleneck. Most subjects are able to spot the second target on at least a small proportion of trials. Since this task gauges the ability of subjects to allocate cognitive resources efficiently when multiple stimuli compete for attention, it is perfectly suited for investigations of the effects of mental training on attention.

Previous studies had reported that the act of meditation can alter cognitive and perceptual abilities and neural responses. However, Davidson and colleagues wondered whether volunteers who received three months of intensive training in a particular type of meditation, known as Vipassana meditation, would allocate attentional resources more efficiently and therefore show enhanced performance on the attentional blink task, a task that taps into similar skills used during training without directly involving meditation. Vipassana meditation encourages “non-reactive awareness”—a state of mind in which individuals cultivate awareness of stimuli without judgments or affective responses to those stimuli.

Since Vipassana meditation allegedly reduces mental distraction, the authors hypothesized that volunteers (“practitioners”) who attended the intensive training course, which involved 10–12 hours of meditation each day, would be more successful at identifying the second target, because the subjects’ attention would be captured less by the first target. Performance on the task before training was compared with performance after training, and also with that of a control group (“novices”) who were interested in meditation but received only one hour of training, and meditated for 20 minutes each day for the week that preceded each experimental session.

**Figure:**
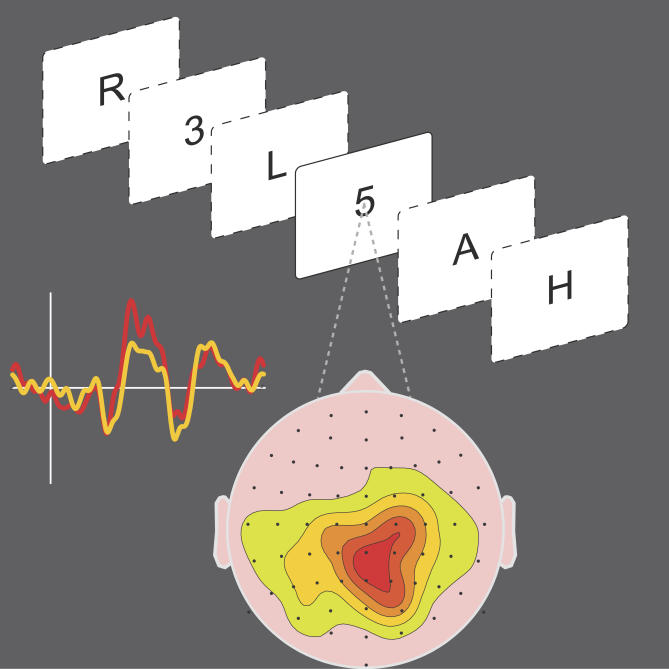
This image depicts the scalp sites where the reduction in brain-resource allocation to the first of two target stimuli (T1) observed after intensive meditative training was significant. The waveforms represent T1-evoked brain potentials as measured at time 1 (red line) and time 2 (orange line).

After the three-month training period, each member of the practitioner group showed improved detection of the second target, if it appeared within half a second after the first target. Only 16 out of 23 of the novice group showed a similar improvement. This reduction in the effect of the attentional blink is consistent with the idea that after training, practitioners were allocating a smaller proportion of their brains’ resources to the first target.

Another way of measuring the allocation of attention is to use event-related potentials—electrical changes associated with neural responses to sensory stimuli or cognitive tasks, which can be recorded through the scalp. When event-related potentials are recorded from subjects during the attentional blink task, a noticeable electrical change—called the P3b—is associated with the appearance of the first target. This event is believed to reflect the allocation of resources to the target. In the practitioner group, after three months of intensive mental training, the P3b that was associated with the first target was significantly smaller for those trials in which the subject was able to identify both targets. In other words, the event-related potentials appeared to show that less attention was being allocated to the first target, and this allowed the subjects to spot the second target.

To investigate further the possible link between attentional resource allocation, as reflected by the size of the P3b potential, and performance on the attentional blink task, the authors compared individual performance on the task with the event-related potentials recorded from each subject. Subjects who showed the largest decrease over time in the size of the P3b evoked by the first target also generally showed the greatest improvement in detection of the second target. This result further corroborates the view that the attentional blink is caused by excessive allocation of attentional resources to the processing of target 1.

Importantly, the subjects did not meditate during the attentional blink task. So these results indicate that intensive mental training can produce lasting and significant improvements in the efficient distribution of attentional resources among competing stimuli, even when individuals are not actively using the techniques they have learned.

